# Recent Advances in Multifunctional Wearable Sensors and Systems: Design, Fabrication, and Applications

**DOI:** 10.3390/bios12111057

**Published:** 2022-11-21

**Authors:** Shigang Jia, Hongwei Gao, Zhaoguo Xue, Xianhong Meng

**Affiliations:** Institute of Solid Mechanics, Beihang University (BUAA), Beijing 100191, China

**Keywords:** multifunctional wearable sensors, health monitoring, function integration, 3D sensor

## Abstract

Multifunctional wearable sensors and systems are of growing interest over the past decades because of real-time health monitoring and disease diagnosis capability. Owing to the tremendous efforts of scientists, wearable sensors and systems with attractive advantages such as flexibility, comfort, and long-term stability have been developed, which are widely used in temperature monitoring, pulse wave detection, gait pattern analysis, etc. Due to the complexity of human physiological signals, it is necessary to measure multiple physiological information simultaneously to evaluate human health comprehensively. This review summarizes the recent advances in multifunctional wearable sensors, including single sensors with various functions, planar integrated sensors, three-dimensional assembled sensors, and stacked integrated sensors. The design strategy, manufacturing method, and potential application of each type of sensor are discussed. Finally, we offer an outlook on future developments and provide perspectives on the remaining challenges and opportunities of wearable multifunctional sensing technology.

## 1. Introduction

Due to attractive advantages in lightweight, portability, conformity, and comfort, flexible electronics have made great progress in theory [1,2] and application [3,4,5,6,7,8] in recent years. As a very important application direction of flexible electronic technology, wearable sensors have also developed rapidly with the progress of miniaturized electronic technology and the enhancement of people’s health awareness [9,10,11,12,13]. In addition, materials with super flexibility and super fold-ability have also been used in flexible electronics [14,15], which will help to further improve the performance of flexible electronic devices. For example, a super-foldable conductive carbon material prepared by Zan et al. [16] can remarkably bear 1,000,000 times repeated true-folding without structural damage and conductivity fluctuations. The earliest use of wearable sensors for human health monitoring can be traced back to 1960 [17]. To realize the Apollo Program, it was necessary to continuously monitor the physiological signals of astronauts, such as heart rate and body temperature, and send the data back to the earth, thus generating a primary wearable sensor that can continuously monitor the health status. After decades of development, wearable sensors have been greatly improved in functional diversity [18,19,20], device miniaturization [21,22,23], and wearing comfort [24,25,26,27,28], and the market demand is also expanding [29]. According to a report by Grand View Research [30], the global wearable sensor market is expected to reach 2.86 billion dollars by 2025, growing at a surprising compound annual growth rate of 38.8% in the forecast period.

**Figure 1:**
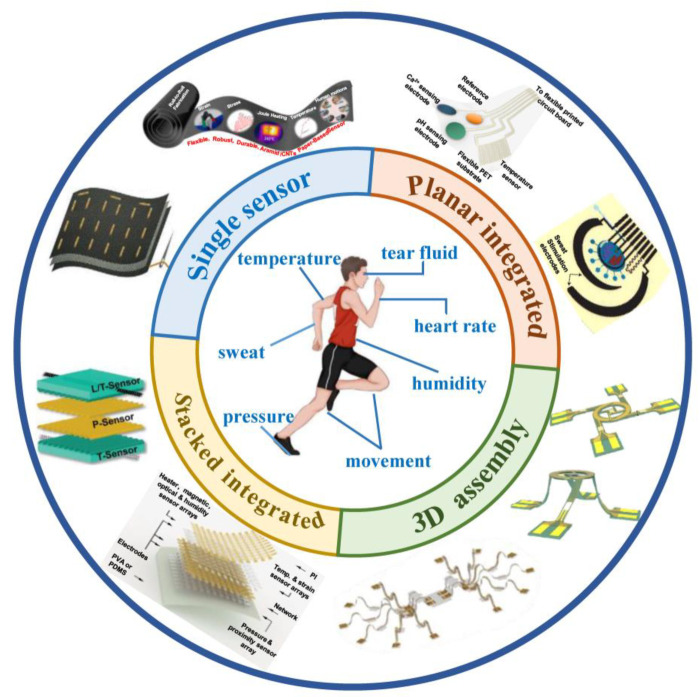
The functionalities of wearable sensors and the modes of function integration. Single sensors with multiple functions: reprinted with permission from [10,77]. Copyright 2021 American Chemical Society. Copyright 2021 Wiley-VCH. Planar integrated: reprinted with permission from [78,79]. Copyright 2016 American Chemical Society. Copyright 2022 Nature Publishing Group. 3D assembly: reprinted with permission from [80,81]. Copyright 2021 PNAS. Stacked integrated: reprinted with permission from [82,83]. Copyright 2017 Wiley-VCH. Copyright 2018 Nature Publishing Group.

The motivation for developing wearable sensors and systems is because of the tremendous benefits associated with real-time and continuous monitoring of the physical and physiological signals of the human body [25,31,32,33,34,35]. These monitoring signals can provide important information for health status assessment [36,37,38], disease diagnosis [39,40,41], and treatment [42]. For example, the size of tumors in the body can be measured in real-time through the strain sensor conformally attached to the skin [43], and blood pressure can be continuously measured using the pressure sensor [44]. However, due to the complexity of human physiological signals, it is often necessary to judge the health status of the human body through multiple information at the same time, which requires wearable sensor systems capable of numerous sensing functions [45,46,47]. Specifically, as shown in Figure 1, the signals that the wearable sensor can monitor include movement [48,49,50], pressure [51,52,53], temperature [54,55,56], humidity [57,58,59], and heart rate [60,61,62]. In addition, through the electrochemical analysis of human secretions (such as sweat [63] and tears [64]), a variety of physiological signals can be obtained [65], such as the concentration of glucose [66,67], lactic acid [68,69], sodium, and potassium ions [70,71,72]. There have been several excellent review articles on the topic of flexible and wearable sensors [73,74,75,76]. They introduced the new materials, fabrication strategies, properties, and applications of flexible sensors and summarized the outstanding contributions of wearable sensors to health monitoring and biomedical fields in detail. These articles will be of great help to scholars who plan to start studying in this field. However, there is a lack of overview written from the perspective of how to achieve multifunctional wearable sensors at present. Our article will supplement this aspect.

In this review, we intend to present an overview of the recent advances in wearable multifunctional sensors and systems for human healthcare, as shown in Figure 1. Section 2 highlights the representative advances of single sensors with multiple functions based on material sensing properties. Section 3 focuses on the recent progress in planar-integrated multifunctional sensors and systems. Section 4 and Section 5 provide an overview of the three-dimensional (3D) layout sensors, including 3D assembly and stacked integration. Finally, conclusions and future outlooks in the field of wearable multifunctional sensors and systems are discussed in the final section.

## 2. Single Sensors with Multiple Functions

Sensor is a measuring device that can convert the measured nonelectric quantity into corresponding electric quantity or electric parameter output that can easily be accurately processed according to a certain rule. Many materials can be used to make sensors, such as semiconductor, ceramic, metal, and organic materials. With the special sensing properties of some materials, multiple functions can be realized in a single device. Table 1 summarizes the materials, manufacturing methods, and sensing performance of several typical single sensors with multiple functions. This section introduces the latest developments in this field.

**Figure 2:**
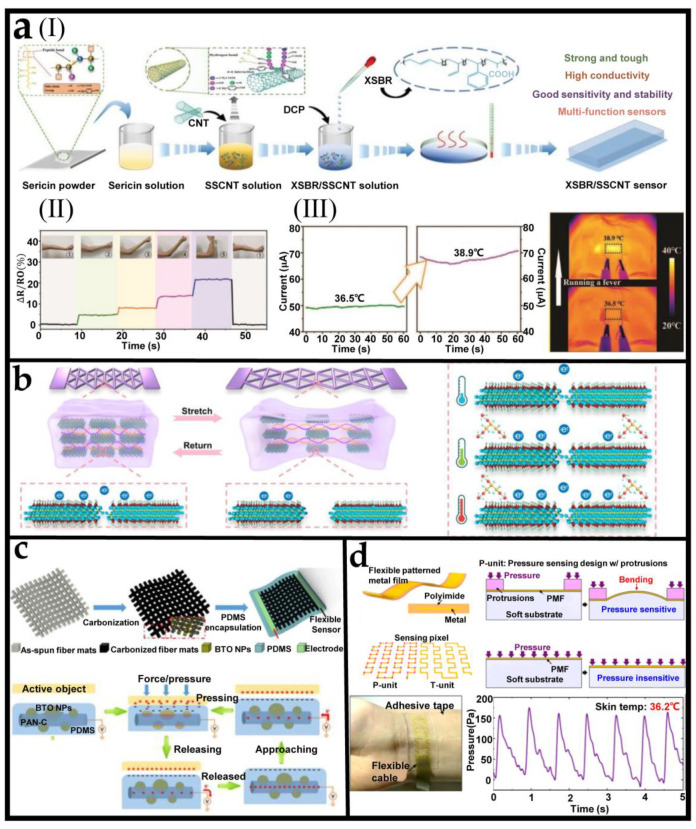
Single sensors with multiple functions. (**a**) (**I**) The preparation of flexible XSBR/SSCNT sensors. (**II**) Bending of the elbow and corresponding signals. (**III**) Measurement of human forehead temperature before and after “artificial fever” with XSBR/SSCNT sensors. Reprinted with permission from [88]. Copyright 2022 Wiley-VCH. (**b**) Strain and temperature sensing mechanisms of the printed conductive hydrogel. Reprinted with permission from [89]. Copyright 2022 Nature Publishing Group. (**c**) The fabrication process and working mechanism of the flexible dual-function PAN-C/BTO self-powered pressure sensor. Reprinted with permission from [90]. Copyright 2018 American Chemical Society. (**d**) The sensing mechanism and applications of the e-skin prototype. Reprinted with permission from [91].

To design and fabricate stretchable multifunctional sensors, a widely used method is to embed functional conductive nanomaterials (e.g., carbon nanotubes (CNTs) [84], graphene [85] and metal nanoparticles [86], etc.,) into elastic material (e.g., rubber, silicon) to form stretchable conductive films. When the conductive film is deformed, or the ambient temperature changes, the resistance of the conductive film will change accordingly. In this method, the uniformity of conductive nanomaterials in rubber has an important impact on the performance of the sensing film [87]. Recently, Lin et al. [88] found that sericin enables the homogeneous dispersion of CNTs in the rubber matrix, and designed a multifunctional sensor based on carboxylic styrene butadiene rubber (XSBR) and hydrophilic sericin (SS) non-covalently modified CNTs. As shown in Figure 2(aI), the conductive rubber film was fabricated by latex film-forming method. A stable aqueous suspension of sericin-CNTs (SSCNT) was made via mixing CNTs powder and sericin solution first, and then the XSBR latex and the dicumyl peroxide (DCP) vulcanizing agent were added slowly into SSCNT aqueous suspension under stirring. Finally, the XSBR/SSCNT mixture was poured into a mold where it was dried until it reaches a constant weight. By attaching the conductive rubber film to the elbow skin, the motion of the elbow can be monitored in real-time (Figure 2(aII)). Moreover, as shown in Figure 2(aIII), when the conductive rubber film was attached to a person’s forehead with normal body temperature (36.5 °C), the device showed a current of about 49.53 µA. Then, when the subject’s temperature rose to 38.9 °C, the response current sharply increased to 67.64 µA. Based on these characteristics, the resultant versatile sensors showed potential applications in serving as wearable sensors for full-range recognition of human activities and measuring body temperature.

Owing to the specific advantages of the intrinsic stretchability, stable conductivity, biocompatibility, and mechanical robustness, conducting polymer hydrogels are widely recognized as promising candidate materials for flexible sensors [85]. Figure 2b shows a multifunctional sensor with excellent strain and temperature sensing performance based on Ti_3_C_2_Tx bonded polymer hydrogel [89]. This hydrogel was prepared through a facile 3D printing strategy using direct ink writing (DIW) technology and solvent replacement. The printed hydrogel can be directly used for sensing without additional encapsulation. The stretchable resistive sensor based on the printed hydrogel performed noticeable strain responses with the maximum GF of 5.7 within 191% strain. In addition, due to the synergy between temperature and tunneling effect, the hydrogel demonstrates terrific temperature-sensitive behaviors with temperature coefficient of resistance (TCR) of −5.27% °C^−1^ (0–30 °C) and −0.84% °C^−1^ (40–80 °C).

As shown in Figure 2c, a carbonized electrospun polyacrylonitrile/BaTiO_3_ (PAN-C/BTO) nanofiber mat was encapsulated in PDMS to form a dual-function sensor [90]. Fabricated by a convenient electrospinning, carbonization, and encapsulation process, the carbonized PAN-C/BTO based sensor could detect curvature based on the impedance change of the conducting nanofiber and detect pressure via the BaTiO_3_ nanoparticle-enhanced singe-electrode triboelectric nanogenerator independently. The bottom panel of Figure 2c shows the pressure-sensing mechanism of this sensor. The carbonized PAN nano-fibers act as electrodes and BaTiO_3_ nanoparticles in nanofibers of PDMS act as a negative triboelectric friction layer. When the active object contacts PDMS, BTO will be poled by structural deformation to generate a local piezoelectric potential. At the same time, tri-boelectrification will also occur at the interface between the active object and PDMS. With the separation of the object from the sensor surface, equivalent positive charge would be induced on the conductive nanofiber layer due to electrostatic induction effect. Owing to the pressure and curvature sensing functions, the nanofiber sensor can be applied to physical detection and human motion sensing. For example, multiple sensors can be integrated into the sole to capture human gait.

Although the multifunctional sensor based on a conductive polymer composite has good extensibility and conductivity at the same time, because the material’s conductivity is sensitive to different factors (strain and temperature), there will be cross interference of multiple sensing signals. To avoid measurement errors caused by sensor information coupling, Cai et al. [91] fabricated a multifunctional e-skin based on patterned metal films (PMFs) for tactile sensing of pressure and temperature with a broad linear response range by implementing the single sensing mechanism of piezoresistivity. As shown in Figure 2d, a sensing pixel consists of a pressure sensing unit (P-unit) with microprotrusions and a temperature sensing unit (T-unit) without microprotrusions. In the P-unit, the spatially distributed microprotrusions on the PMF switch the negligible out-of-plane compression of PMFs due to pressure to the appreciable bending deformation, which enables the P-unit to be pressure sensitive and leads to a measurable resistance change reflecting the magnitude of the pressure. In the T-unit, the pressure induces negligible deformations in the PMF such that the T-unit is pressure insensitive, and temperature could be detected by measuring the resistance change of the T-unit without the interference of pressure. When the multiple stimuli of temperature and pressure are applied to the sensing pixel, the resistance change of the P-unit is caused by both pressure and temperature, while that of the T-unit is only caused by temperature. The resistance change of the T-unit then yields the sensing signal of temperature, which could be used to eliminate the temperature interference on the P-unit, thus yielding the accurate sensing signal of pressure. The multifunctional e-skin can monitor pulse and swallowing, and can also be attached to the flexible gripper to feel the softness and temperature of objects. It is worth noting that the manufacturing method of the e-skin is fully compatible with the mature micro-manufacturing process, which makes the batch manufacturing of this flexible multifunctional electronic skin feasible.

## 3. Planar-Integrated Multifunctional Sensors

Continuous monitoring of a person’s health data, such as heart rate, body temperature, and blood pressure, will help medical care providers predict and diagnose dangerous conditions or diseases early so that patients can get faster and more effective treatment. Therefore, it is of excellent application significance to develop multifunctional wearable sensors and systems capable of monitoring various complex physiological signals for patients. Although the sensors based on a single device (or material) can also sense two or more kinds of information, they still struggle to decouple multiple complex physiological signals. Integrating various high-performance sensors in a plane and performing their respective functions is feasible. This section highlights the recent progress of these planar-integrated multifunctional sensors.

**Figure 3:**
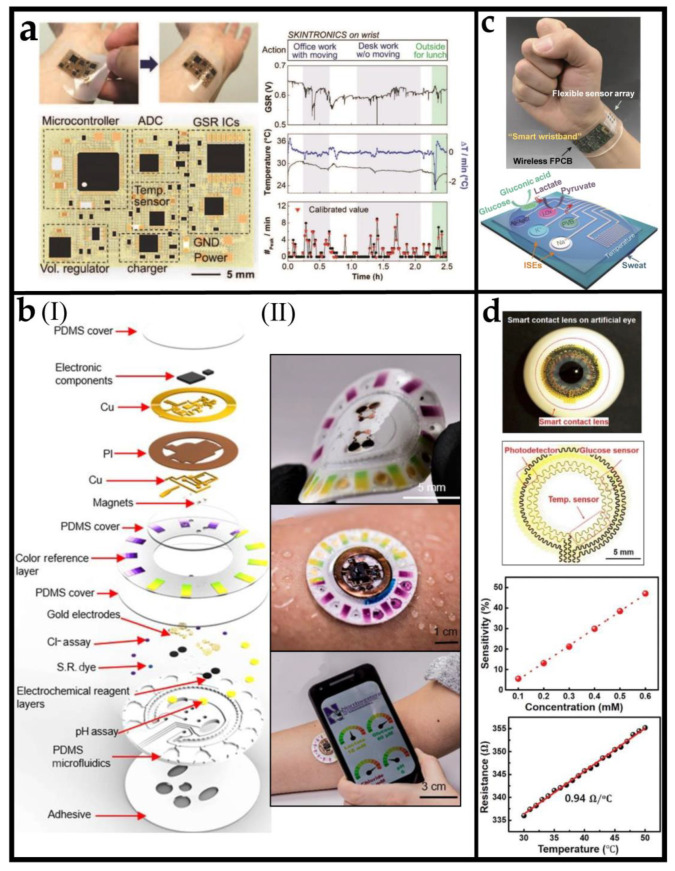
Planar integrated multifunctional sensors: (**a**) The stretchable circuit and real time monitoring results of kin-like bioelectronics (SKINTRONICS) for stress monitoring. Reprinted with permission from [92]. Copyright 2020 Wiley−VCH. (**b**) (**I**) Schematic illustrating the exploded view of the complete hybrid battery-free system. PI, polyimide; S.R., sweat rate. (**II**) Image illustrating the device and a phone interface that illustrates wireless communication and image acquisition. Reprinted with permission from [96]. (**c**) Images and schematic illustrations of the FISA for multiplexed perspiration analysis. Reprinted with permission from [97]. Copyright 2016 Nature Publishing Group. (**d**) The glucose sensor’s structural design, sensitivity (|DR|/R0) and temperature-dependent resistance curve. Reprinted with permission from [98]. Copyright 2021 Elsevier Inc.

Figure 3a shows a fully integrated, stretchable, wireless skin-conformal bioelectronic. Integrating soft, nanomembrane sensors and electronics allow continuous and portable stress monitoring in daily life [92]. Stretchable nanomembrane electrodes and a digital temperature sensor enable highly sensitive monitoring of galvanic skin response (GSR) and temperature. This device consists of a pair of skin-conformal thin-film sensors and a stretchable membrane wireless circuit, together integrated on a soft elastomeric membrane. The device fabrication utilizes multiple sequential techniques, including a microfabrication process to construct necessary patterns with photolithography, metallization, transfer printing, and integration of chip components. The combination of GSR and temperature sensors removes any unwanted signal fluctuation caused by skin-temperature change. At the same time, the wireless, intimate contact of the entire device on the skin provides negligible motion artifacts. The sensor attached to the wrist can continuously monitor the pressure level by calculating the number of GSR peaks per minute and temperature compensation.

For human health monitoring, it is important to monitor the changes of key metabolites, because they usually reflect the symptoms of major diseases and warn us of potential health risks. As a representative biofluid, sweat is of particular interest owing to its relative ease of noninvasive collection and its rich content of electrolytes and small molecules. The selection of biomarkers is crucial to the results of health monitoring. Target analytes in sweat sensor are mainly selected based on their informative role in understanding an individual’s physiological state. For example, sweat lactate can serve as a sensitive marker of pressure ischemia [93], sweat glucose sensing can potentially serve as a non-invasive way for blood glucose monitoring [94], and sweat chloride provides important insights into electrolyte imbalance [95]. By combining the chronometric microfluidic platforms and embedded colorimetric assays, Bandodkar et al. [96] fabricated a battery-free wireless electronic sensor platform that can simultaneously monitor sweating rate/loss, pH value, lactic acid, glucose, and chloride. Figure 3(bI) shows the exploded view illustration of the overall construction of each sub-system. The platform comprises a disposable soft microfluidic network and reusable thin near-field communication (NFC) electronic modules. As shown in Figure 3(bII), the soft and flexible construction allows comfortable, water-tight, and irritation-free mounting onto curved regions of the body. The NFC interface supports both wireless power delivery and data transmission to any NFC-enabled consumer device, such as a smartphone, tablet, or watch. Moreover, by comparing the glucose and lactic acid levels in sweat collected by the sensor platform with the levels in the blood, it was found that there was a correlation between the two, which indicated that the device had the potential to track the concentration of blood analyte in a noninvasive manner.

Due to the complex compositions of sweat, it is still challenging to detect a plurality of biomarkers using a single wearable device and perform real-time signal processing to evaluate health status accurately. Remarkably, Gao et al. [97] demonstrated a wearable, flexible integrated sensing array (FISA) for multiplexed in situ perspiration analysis, which realizes simultaneous and selective measurement of abundant sweat biomarkers (such as glucose, lactate, sodium, and potassium ions) and skin temperature, as present in Figure 3c. At an unprecedented level of integration, this work merged various functionalities, including signal transduction, conditioning, processing, and wireless transmission, into a single flexible system for real-time monitoring of subjects’ physiological states. It comprises skin-interfaced plastic sensors (five different sensors) and silicon integrated circuits consolidated on a flexible board (more than ten chips). In addition, for the accuracy of the measurement results of various biomarkers in sweat, a signal processor is built in to compensate for and eliminate the impact of temperature in the reading of chemical sensors. By wearing the FISA on different body parts, such as wrists, arms, or forehead, the lightweight and comfortable wearable sensor can be applied for prolonged indoor and outdoor activities, allowing the real-time assessment of health status.

In addition to sweat-sensing devices, the design of planar-integrated sensors has also been applied in tear detection. Clinical trials have demonstrated clearly that tear fluid can be a valuable marker for the measurement of systemic glucose [99]. This innovative contact lenses equipped with high-sensitivity glucose sensors could open the possibility of a non-invasive method to detect biomarkers in tears continuously. Figure 3d shows multifunctional intelligent contact lenses with an ultrathin MoS_2_ transistors-based serpentine mesh sensor system [98]. The integrated sensor systems contain a photodetector for receiving optical information, a glucose sensor for monitoring glucose levels directly from tear fluid, and a temperature sensor for diagnosing potential corneal disease. The serpentine mesh sensor system can be directly mounted onto the lenses and maintain direct contact with tears, delivering high detection sensitivity, while being mechanically robust and not interfering with either blinking or vision. The capabilities of the devices include detection of optical signals, glucose levels, and corneal temperature. These results provide not only an alternative solution for manufacturing advanced smart contact lenses but also a novel insight into designing other multifunctional implantable bioelectronics. Compared with sweat sensor, the integrated contact lens sensor system has the advantage of continuously detecting biomarkers without additional exercise. However, it has the disadvantage of complex wearing, and the narrow space on the contact lens also limits the integration of more functions.

## 4. 3D Assembled Multifunctional Sensors

3D assembly (e.g., rolling, folding, curving, and buckling) has received extensive attention in recent years [100,101,102]. Transforming 2D thin films into complex 3D structures could be compatible with well-established planar micromanufacturing technologies and rearrange function materials/components in a 3D layout to expand novel functions, enhance performance, and improve integration. These 3D assembly methods have demonstrated new routes for designing and fabricating various novel 3D wearable sensors [103,104]. In this section, we focus on the representative advances in this field of 3D assembled multifunctional sensors.

**Figure 4:**
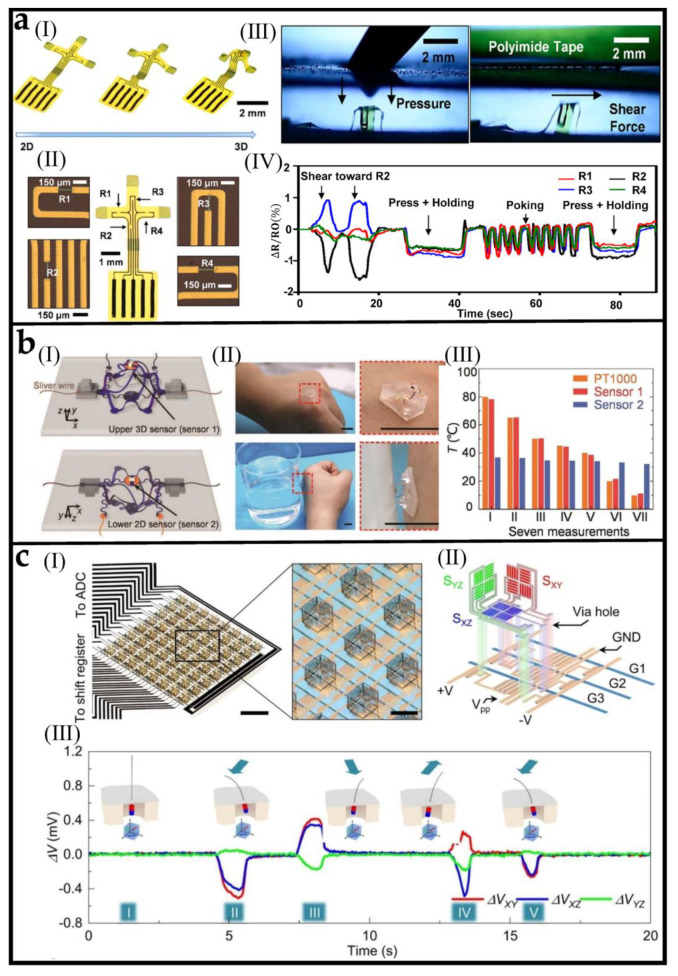
3D assembled multifunctional sensors (**a**) (**I**) 3D microelectromechanical sensor molding diagram. (**II**) Optical microscope images of 2D precursor that have four piezoresistors. (**III**) Sensor responses to normal force (left) and shear force (right). (**IV**) The sensor recorded response to shear force and normal pressure. Reprinted with permission from [105]. Copyright 2019 American Chemical Society. (**b**) (**I**) Schematic diagram of 3D sensor composed of sensor 1 and sensor 2. (**II**) Optical image of water temperature measurement. (**III**) Electrical responses of the sensor to seven different water temperatures. Reprinted with permission from [106]. Copyright 2021 Wiley−VCH. (**c**) (**I**) Optical image of an IMOS device and the image of the pixel array in the device. (**II**) Exploded schematic and circuit diagram of a single pixel. (**III**) Single pixel response over time by bending the hair in different directions. Reprinted with permission from [107]. Copyright 2022 Nature Publishing Group.

Figure 4(aI) shows a 3D tactile sensor generated by mechanically guided geometric transformation of a 2D planar precursor [105]. The 2D precursor consists of four rectangular silicon nanomembrane (Si-NM) piezoresistors (denoted as R1, R2, R3, and R4) as shown in Figure 4(aII). Compared with the plane structure, the sensor can sense the de-formation of space after forming a 3D structure, and responses of each of the piezoresistive elements configuration yield separate electrical outputs that could independently characterize the stress tensor associated with applied force in Figure 4(aIII). When the pressure and shear force are applied simultaneously, the changes in four resistances correspond to the linear superposition of those when the load is applied separately and, therefore, can determine the pressure and shear force simultaneously. Figure 4(aIV) shows the response of the 3D sensor wirelessly recorded sensing data. The shear force and normal pressure can be decoupled. This strategy that reproduces the complex characteristics of skin receptors in the skin provides an effective idea for the design of sensors [105].

Figure 4(bI) shows a 3D flexible Janus temperature sensor. The top surface of the elastic substrate is a 3D temperature sensor (sensor1). The 3D structure is formed by fixing two bonding points of the 2D precursor to the prestrained substrate and then releasing the prestrained substrate. The bottom surface of the elastic substrate is a 2D temperature sensor (sensor2). Owing to the low thermal conductivity of air, this sensor can realize the decoupling measurement of skin temperature and target object temperature. The full anti-fatigue design makes the resistance change of both sensors less than 0.2% after 10^5^ cycles of testing. Figure 4(bII) shows the temperature sensor attached to the hand to measure the temperature of cold/hot water. Figure 4(bIII) shows the measurement results of seven different water temperatures. It can be seen that sensor 1 and sensor 2 can achieve good decoupling (PT1000 is the result measured by a commercial barshaped thermocouple) [106].

Folding (or origami) assembly, folding the relative rigid panels by pre-defined angles through localized bending deformations, is also an effective method for fabricating 3D sensors. Figure 4(cI) shows the active matrix-integrated micro-origami magnetic sensor (IMOS) device which are realized by autonomous folding of photolithographically defined planar microstructures into cubic architectures [107]. Each pixel contains three magnetic bridges located on the orthogonal planes of the cube (Figure 4(cII)). The increased dimension makes it possible to detect the magnetic field’s vector characteristics fully. Furthermore, the IMOS device is integrated with a flexible composite skin layer and embedded magnetic hair, the movement of the hair in all directions can also be detected, as shown in Figure 4(cIII).

The mechanically guided 3D assembly method is a powerful means to form sophisticated 3D microstructures. It is challenging to batch manufacture high-performance inorganic electronics with complex 3D configurations according to traditional processing methods. Even advanced manufacturing methods such as 3D printing cannot escape the restriction of material types and processing efficiency. As the 3D assembly method is obtained from the morphological transformation based on 2D precursor, it is fully compatible with advanced plane micromanufacturing technologies. The guidance of basic mechanical theory makes 3D assembly technology have versatility. With wearable sensors’ increasingly complex service scenarios, new methods are urgently needed to expand their adaptability and functionality. The 3D assembly method provides an important route to develop new multifunctional wearable sensors.

## 5. Stacked Integrated Multifunctional Sensors

Large stretchability and high functional density (or area coverage ratio) are required for some wearable sensing applications; however, these two key performance indicators often restrict each other in high-performance flexible inorganic electronics [108,109]. Therefore, to achieve larger stretchability to accommodate the large deformation of some parts of the human body, the area coverage of functional components has to be sacrificed for single-layer equipment, which limits the performance of wearable sensors due to the reduced functional density. Recently, a series of 3D multilayer integration strategies have been proposed, making it possible to achieve both large stretchability and a high area coverage ratio (even >100%), which is impossible in single-layer integration.

**Figure 5:**
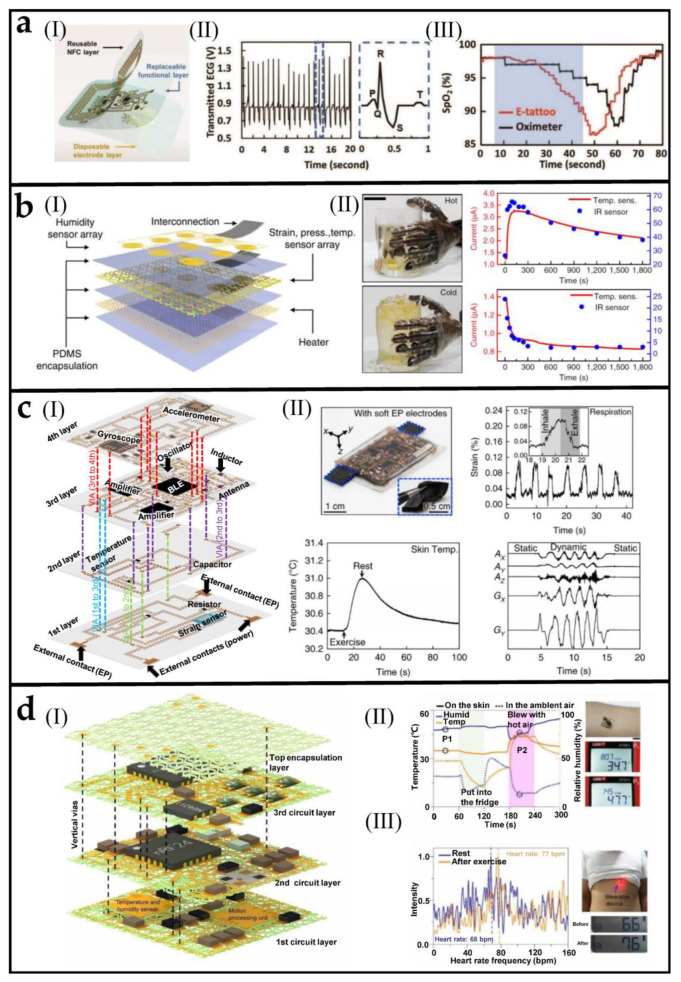
Stacked integrated multifunctional sensors. (**a**) (**I**) Schematic of the modularized e-tattoo. (**II**) Wirelessly measured ECG by the e-tattoo. (**III**) SpO2 measured wirelessly by the e-tattoo (red) and by a commercial oximeter (black) before. Reprinted with permission from [110]. Copyright 2019 Wiley−VCH. (**b**) (**I**) An exploded view of the artificial skin comprised six stacked layers. (**II**) Images of the prosthetic limb touching a cup of hot and iced water. Reprinted with permission from [111]. Copyright 2014 Nature Publishing Group. (**c**) (**I**) Exploded schematics of the system fabricated layer by layer. (**II**) An optical image of the system integrated with CNT/silicone and the output signals of strain, temperature, and acceleration. Reprinted with permission from [112]. Copyright 2018 Nature Publishing Group. (**d**) Design and fabrication of densely packed, a stretchable electronic system based on SMNM and demonstration of functional characteristics of the SMNM-based electronic system (**I**) Schematic illustration of the device system in an exploded view. (**II**) Temperature and relative humidity measured by SMNM-based electronic system. (**III**) Results of heart rates extracted from the frequency analysis of accelerometer data, obtained from the device. Reprinted with permission from [113].

Figure 5(aI) shows a stacked integrated multifunctional wearable device which is manufactured by a rapid, freeform “cut-solder-paste” method introduced in the original text [110]. Each layer is modularized and supports disassembly and reuse. The waste caused by discarding the whole device after a single use is avoided. The top NFC layer (integrated access communication device) and the middle functional layer (integrated functional sensor device) can be reused, while the electrode layer directly in contact with the skin can only be used once. Through the assembly, a thin electronic tattoo can be made to measure physiological signals such as electrocardiogram and blood oxygen saturation (Figure 5(aII,III)).

The multifunctional sensors integrated by stacking can also be made into electronic skin and attached to the surface of the prosthetic. Figure 5(bI) is a schematic diagram of each layer stacked, integrating the heater, strain, temperature, pressure sensor, and humidity sensor [111]. The sensor network attached to the artificial hand has a spatially variable geometric design to adapt to the geometric and deformation characteristics of different hand positions. In addition to supporting the measurement of temperature, humidity, and pressure signals, the prosthetic hand can simulate the output of human hand temperature due to the integrated heater. Figure 5(bII) shows that once the prosthetic hand touches a cup of hot/ cold water, it can successfully conduct temporary temperature monitoring (red). An IR sensor measures the control temperature (blue). It also reflects the sensor’s ability to respond promptly and accurately.

Figure 5(cI) presents a four-layer stacked sensor’s structure and circuit design [112]. Vertical interconnect accesses (VIAs) for interlayer electrical connections are formed from conductive fillings in craters in silicone. A reasonable functional component layout achieves a high integration degree, making the whole system have a Bluetooth communication function and realize the simultaneous collection of strain, temperature, acceleration, and various physiological signals. The top left panel of Figure 5(cII) shows an optical image of this four-layer stretchable device system. Attaching it to the chest can record people’s respiratory status, body temperature, and exercise status at the same time. The strain sensor can detect the movement of the chest when people breathe. When the human body is moving or resting, obvious changes in skin temperature are captured by temperature sensors. Human motion can be captured by an accelerometer.

The stacked integrated design strategy can achieve high coverage area ratio of the functional component. However, adopting a solid-state encapsulation strategy will highly limit the overall stretch of electronic devices. Due to space constraints, serpentine interconnection cannot play its ultra-high tensile function. A novel method was developed to use multilayer soft network materials as a common platform and the stacking design of encapsulation materials [113]. Multilayer soft network materials are composed of periodic triangular lattices of horseshoe-shaped microstructures. Add silver pillars into the pre designed and manufactured vertical vias (highlighted by dashed lines), followed by soldering, to connect the adjacent layers electrically and mechanically as shown in Figure 5(dI). Under tensile load, the serpentine interconnection can release the total strain energy through out-of-plane deformation. This cellular encapsulation does not constrain the deformation of the serpentine interconnections, thereby enabling a substantial enhancement of the elastic stretchability (about 7.5 times) compared to that with solid encapsulation. In addition, network materials have good air permeability to improve wearing comfort. This highly integrated and miniaturized multifunctional device can accurately collect temperature, relative humidity, and the change in heart rate when the human body moves, as demonstrated in Figure 5(dII,III).

## 6. Conclusions and Perspective

This review presents an overview of the recent advances in four types of multifunctional wearable sensors and systems, i.e., single sensors with multiple functions, planar integrated multifunctional sensors, 3D assembled multifunctional sensors, and stacked integrated multifunctional sensors. Each type has pros and cons, and a trade-off between performances is still necessary. A simple comparison of the four types of sensors is shown in Table 2. The single sensors with multiple functions are usually realized by composite materials and designed structures such as conductive polymers, which have both good conductivity and stretchability. Because the conductivity of materials is affected by strain, temperature, or other factors, the sensors made of them can also sense multiple signals. This type of multifunctional sensor has the advantages of low cost and simple fabrication. But the coupling of multiple signals will affect the measurement results. While some work has reported that sensing signals can be decoupled by some ingenious structural design, the types of sensing functions that can be realized by this method are still very limited. Planar integration is an effective and easy way to solve this problem. By integrating different sensor components onto one layer of flexible substrate and connecting the sensors with interconnects, the sensing device can detect multiple signals simultaneously. However, its level of integration is limited by the density that can be achieved by the manufacturing process of individual devices and circuits. The integration approach to the 3D layout can further improve the level of integration. The 3D assembly method can be compatible with advanced planar micromanufacturing technologies. Rearranging functional materials/components in 3D structures can expand novel functions, enhance performance, and improve integration. As an emerging class of methods, 3D assembly is still not mature and complete, and needs to be further developed. For example, developing a complete theoretical system and effective inverse design method that can accurately guide the assembly of arbitrarily sophisticated 3D configurations is required, and there is still a lack of mature encapsulation strategy for 3D assembly structures. The stacked integrated design strategy has great potential for high-density functional integration, which develops the space in the thickness direction. Of course, it must be admitted that the increase in integration also inevitably increases the thickness and reduces flexibility. Moreover, the reliable electrical and mechanical connection between layers puts forward high requirements for design and manufacturing.

Despite this significant set of progress, many challenges and opportunities remain in the field of wearable sensors and systems. First, although the function integration can be significantly improved through the 3D integration strategy, the manufacturing is complicated and there are still many challenges away from mass production and application. How to improve the yield and success rate is the direction to be broken through in the future. Second, encapsulation is essential for wearable sensors to meet the requirements of security and stability. However, the existing solid encapsulation strategy significantly limits the stretchability. It is a possible solution to suppress the mechanical interaction between the encapsulation layer and inorganic devices/interconnects through new materials and structural design. In addition, the correlation analysis of various sensing information is still insufficient. By introducing tools such as machine learning and artificial intelligence, more intelligent wearable systems will be developed to analyze multiple physiological signals to comprehensively and accurately evaluate human health. They will provide fundamental support for wearable sensors in daily health monitoring, precision medicine, and other fields. Finally, compatible with low-cost mass manufacturing, it is still a big challenge to develop high density, high sensitivity, lower power consumption, high robustness, and complex signal detection of flexible wearable electronic sensors.

## Figures and Tables

**Table 1:** Summary of single sensors with multiple functions.

Authors	Materials	Preparation Methods	Measured Quantities	Detection Range	Maximum Sensitivity
Lin et al. [88]	CNTs, Rubber	Latex film-forming method	Strain	1–217%	Gauge factor 25.98
			Temperature	30 °C–100 °C	0.01636 °C^−1^
Liu et al. [89]	MXenes, Hydrogels	3D printing of direct ink writing (DIW)	Strain	0–191%	Gauge factor 5.7
			Temperature	0 °C–80 °C	−5.27% °C^−1^
Zhao et al. [90]	Nanofiber films	Electrospinning, Carbonization and Encapsulation	Pressure	0.15–25 N	1.44 V·N^−1^
			Curvature	58.9 deg–120.2 deg	1.12 deg^−1^
Cai et al. [91]	Metal, Polyimides	Microfabrication processes	Pressure	0–80 kPa	~0.02 kPa^−1^
			Temperature	0 °C–60 °C	0.083% °C^−1^

**Table 2:** Comparison of four types of multifunctional sensors.

Structure Type	Features	Advantages	Disadvantages
Single sensors	Single sensor with multiple functions	Simple fabrication Low cost	Difficulty in decoupling multiple physical signals
Planar integrated	Multiple sensors arranged in the plane	Independent sensing components Mature fabrication technology Simple design	Low integration
3D assembled	Transform from 2D layout integration to 3D configuration	Novel functions Enhanced performance High integration	Lack of mature and feasible 3D encapsulation methods/strategies
Stacked integrated	Multi layers integration of multiple electronic components	High integration density	Reduced flexibility Fabrication difficulty High cost

## Data Availability

The data presented in this study are available on request from the corresponding author.
